# Microparticles as Potential Mediators of High Glucose-Induced Renal Cell Injury

**DOI:** 10.3390/biom9080348

**Published:** 2019-08-06

**Authors:** Sreenithya Ravindran, Mazhar Pasha, Abdelali Agouni, Shankar Munusamy

**Affiliations:** 1 Department of Pharmaceutical Sciences, College of Pharmacy, QU Health, Qatar University, P.O. Box 2713, Doha 2713, Qatar; 2 Department of Pharmaceutical and Administrative Sciences, College of Pharmacy and Health Sciences, Drake University, Des Moines, IA 50311, USA

**Keywords:** microparticles, diabetic nephropathy, mTOR, ERK1/2, endoplasmic reticulum stress, TGF-β, epithelial-mesenchymal transition

## Abstract

Diabetic nephropathy (DN) is the most common cause of chronic kidney disease worldwide. Activation of signaling pathways such as the mammalian target of rapamycin (mTOR), extracellular signal-regulated kinases (ERK), endoplasmic reticulum (ER) stress, transforming growth factor-beta (TGF-β), and epithelial-mesenchymal transition (EMT), are thought to play a significant role in the etiology of DN. Microparticles (MPs), the small membrane vesicles containing bioactive signals shed by cells upon activation or during apoptosis, are elevated in diabetes and were identified as biomarkers in DN. However, their exact role in the pathophysiology of DN remains unclear. Here, we examined the effect of MPs shed from renal proximal tubular cells (RPTCs) exposed to high glucose conditions on naïve RPTCs in vitro. Our results showed significant increases in the levels of phosphorylated forms of 4E-binding protein 1 and ERK1/2 (the downstream targets of mTOR and ERK pathways), phosphorylated-eIF2α (an ER stress marker), alpha smooth muscle actin (an EMT marker), and phosphorylated-SMAD2 and nuclear translocation of SMAD4 (markers of TGF-β signaling). Together, our findings indicate that MPs activate key signaling pathways in RPTCs under high glucose conditions. Pharmacological interventions to inhibit shedding of MPs from RPTCs might serve as an effective strategy to prevent the progression of DN.

## 1. Introduction

Diabetic nephropathy (DN) is a major complication of diabetes, which affects about 30% to 40% of the patients with type 1 and type 2 diabetes, and ultimately leads to end-stage renal disease (ESRD) [1,2]. Diabetes-induced structural alterations to the kidney such as glomerular basement membrane thickening, glomerular hyperfiltration [3], and tubulointerstitial fibrosis [4] affect the renal function and lead to micro- and macroalbuminuria (leakage of albumin in the urine). DN is highly evident in stage 3, i.e., when a patient is diagnosed with microalbuminuria (30–299 mg/day) followed by macroalbuminuria (>300 mg/day) at stage 4. Renal function is entirely compromised when the glomerular filtration rate (GFR) falls below 15 mL/min (stage 5 or ESRD), which necessitates the need for routine dialysis or kidney transplant for patient survival [5]. Thus, characterization of early biomarkers of DN and development of therapeutic strategies that diminish the severity of DN [6] would prevent the progression of DN to ESRD, and decrease mortality in patients with diabetes. Renal proximal tubular cells (RPTCs) are one of the prominent targets of hyperglycemia-induced injury in DN [7]. Glucose reabsorption by RPTCs follows the tubuloglomerular feedback mechanism, which regulates the GFR [8]. Under hyperglycemic conditions, there is a three-fold increase in the glucose and sodium reabsorption by RPTCs, which leads to glomerular hyperfiltration [9,10,11,12]. The excessive build-up of glucose augments the intracellular glucose efflux within the RPTCs and triggers the secretion of various cytokines, growth factors, reactive oxygen species, and matrix proteins by the RPTCs [7]. This leads to tubular hypertrophy and tubular basement membrane thickening, the two characteristic features of DN [13], and consequently, renal dysfunction in patients with diabetes [2,7].

Microparticles (MPs) are microvesicles, or submicron vesicles shed from cellular plasma membrane upon various stimuli and their size range between 0.1 to 1 µm [14,15]. MPs are released when cells lose asymmetry of the distribution of phospholipids in the plasma membrane, with the most critical change being the externalization of negatively charged phosphatidylcholine causing the formation of blebs, a process called exocytosis. MPs encompass bioactive components such as mRNAs, microRNAs, DNAs, bioactive lipids, and cytokines, and hence, their molecular content reflects the state of activation of their parental cells [16,17]. With regards to DN, accumulating evidence from multiple studies in vitro and in vivo suggest that MPs play an important role in cell-to-cell communication [17,18,19,20,21,22,23,24]. For example, Burger et al. showed that exposure to high glucose stimulated the formation of MPs with pro-oxidative activity from cultured endothelial cells within 24 h and revealed that MPs derived from glomerular podocytes during diabetes serve as early urinary biomarkers in DN [18,20]. Also, recent clinical studies reported higher circulating levels of various types of MPs derived from platelets, leukocytes, and endothelial cells in patients with DN as compared to nondiabetic subjects and in various conditions associated with insulin resistance such as obesity and metabolic syndrome [14,15,19]. However, the exact role of MPs in the pathogenesis of DN is poorly understood. 

Mammalian target of rapamycin (mTOR) and extracellular signal-regulated kinases (ERK) are serine/threonine protein kinases, which not only play an important role in regulating cellular processes such as cell growth, proliferation, and survival but also in the progression of DN [25,26]. The ERK signaling pathway is involved in increasing mRNA translation and ribosomal biogenesis under hyperglycemic conditions in the RPTCs and causes a rapid increase in matrix proteins, thus leading to tubular basement membrane thickening in DN [27,28,29]. Endoplasmic reticulum (ER) is an essential cellular organelle that helps in efficient folding and assembling of proteins post-translation [30]. Under hyperglycemic conditions, the excessive production of proteins overwhelms the ER protein folding machinery and induce ER stress, which contributes to renal cell injury and the progression of DN [31]. Transforming growth factor-beta (TGF-β) has been characterized as the primary mediator in renal fibrosis [32], followed by tubulointerstitial injury under hyperglycemia and initiation of epithelial-mesenchymal transition (EMT) in mature tubular epithelial cells, which is one of the characteristic features of DN [33,34,35,36,37,38]. 

With respect to the involvement of MPs in the progression of DN, Munkonda et al. [21] showed that incubation of RPTCs with MPs derived from podocytes subjected to high glucose-induced stress stimulates profibrotic signaling in renal tubular cells [21]. Similarly, Zhou et al. [22] demonstrated that microvesicles harvested from RPTCs subjected to TGF-β1-induced stress triggers EMT in naïve RPTCs [22]. However, the effects of MPs derived from RPTCs in response to high glucose-induced stress that occurs with DN on naïve RPTCs remain largely unknown. Therefore, in this study, we investigated the paracrine (local) effects of MPs shed by RPTCs in response to high glucose-induced stress on the cell viability, cell cycle progression, and various cell signaling pathways involved in the renal cell injury such as mTOR, ERK, ER stress, TGF-β, and EMT signaling in naïve RPTCs. 

## 2. Materials and Methods 

### 2.1. Cell Culture

NRK-52E cells (Public Health England, London, UK), a rat renal proximal tubule cell line, were cultured in 100 mm culture dishes in Dulbecco’s modified Eagle medium (DMEM; Gibco, Grand Island, NY, USA) supplemented with 5% fetal bovine serum (FBS; Gibco), 1% amino acid supplement (*L*-Glutamine; Gibco), and 1% penicillin/streptomycin (Gibco). A premium grade FBS, which is subjected to triple 0.1 micron filtration, was used in this study. In addition, the complete culture media (after the addition of FBS) was filtered using a 0.2 micron sterile filter to remove any extraneous microparticles introduced by FBS into the media. The cells were grown at 37 °C in a humidified atmosphere of 5% CO_2_ and 95% air. Cells were either cultured on 48-well plates (for cell viability studies) or 6-well plates (for Western blotting and cell cycle analysis) in 5% FBS-supplemented DMEM. All the treatments were done on about 60–70% confluent cells of passage ranging from 7 to 19. 

### 2.2. Isolation of Microparticles (MPs) from RPTCs

NRK-52E cells (RPTCs) were divided into three treatment groups: (1) Control, (2) intermittent high glucose treatment, and (3) continuous high glucose treatment. RPTCs in the control and continuous high glucose-treated groups were exposed to 5% FBS supplemented DMEM with 5 mM glucose and 30 mM glucose, respectively, for 72 h, and their media was replenished every 24 h. To mimic the fluctuations in glucose levels that are typically seen in patients with diabetes, RPTCs in the intermittent high glucose-treated group were exposed to 5% FBS supplement DMEM with 30 mM glucose for 16 h and 5 mM glucose for 8 h, and the cycle was repeated for 72 h. At the end of 72 h, the media from each group were collected for the isolation of MPs as described previously [39,40]. Briefly, the collected media was first centrifuged at 1500× *g* for 10 min at 4 °C to remove cell debris. The supernatant was transferred to 1.5 mL microcentrifuge tubes and centrifuged at 21,000× *g* for 45 min at 4 °C. The supernatant was discarded carefully without dislodging the pellet, and the pellet was resuspended in sterile phosphate-buffered saline (PBS), pH 7.4 (Gibco). The process of centrifugation at 21,000× *g* for 45 min at 4 °C and resuspension in PBS was repeated twice, and the final pellet was suspended in 100 µL of PBS and stored at 4 °C [39,40].

### 2.3. Treatment of Naïve RPTCs with MPs

About 6–7 × 10^4^ RPTCs were added to each well of a 6-well plate containing 5% FBS supplemented DMEM and cultured for 24 h to obtain 50–60% confluency. Then, 10 µg/mL of MPs isolated from the three treatment groups were added to the naïve RPTCs in each well and treated for a further 24 h as previously done by us [39,40]. Cells were then processed based on the assay protocols described below. 

### 2.4. Cell Viability Assay

The viability of naïve RPTCs following 24 h treatment with MPs was measured quantitatively by Alamar Blue^TM^ cell viability reagent (Thermo-Fisher Scientific, Eugene, OR, USA). Alamar Blue^TM^ gets oxidized from a blue color to pink color compound that gives fluorescence in the presence of viable cells. The fluorescence was measured using SpectraMax Plate Reader (Molecular Devices, San Jose, CA, USA) at an excitation wavelength of 544 nm and an emission wavelength of 590 nm. Fluorescence values were normalized to control and expressed as the percentage of control.

### 2.5. Cell Cycle Analysis

Following 24 h exposure to MPs, RPTCs were scraped and stained with propidium iodide solution as described in the manufacturer protocol for Tali^TM^ cell cycle kit (Thermo Fisher Scientific, USA). Cells were incubated in the dark for 30 min and then analyzed for cell cycle progression using the Tali^TM^ image-based cytometer (Thermo Fisher Scientific, Paisley, UK) as described previously [41]. 

### 2.6. Nuclear Extraction

Nuclear extraction was performed according to instructions in the manufacturer protocol for the Nuclear Extraction Kit (Abcam, Cambridge, UK). RPTCs were seeded in 100 mm cell dishes. The cells were harvested with Trypsin-EDTA (0.25%) (Thermo Fisher Scientific, USA) and centrifuged at 1000× *g* for 5 min. The resulting cell pellet was suspended in 100 µL of pre-extraction buffer per 10^6^ cells and transferred into a microcentrifuge tube. The suspension was incubated on ice for 10 min and then vortexed vigorously for 10 s. Upon further centrifugation at 12,000× *g* for 1 min at 4 °C, the supernatant containing cytoplasmic contents was carefully transferred to a new microcentrifuge tube and stored at –20 °C until analysis. The pellet containing nuclear fraction was resuspended in dithiothreitol (DTT) solution containing a protease inhibitor cocktail (1:1100). This mixture was incubated on ice for 15 min with continuous vortexing (5 s) every 3 min. The mixture was centrifuged at 14,000× *g* for 10 min at 4 °C, and the supernatant containing nuclear fraction was transferred to a new microcentrifuge tube and stored at –20 °C until analysis. The isolated cytoplasmic and nuclear supernatants were analyzed for specific proteins by Western blotting. 

### 2.7. Western Blotting

Following 24 h exposure to MPs, RPTCs were scraped and lysed in 0.5 M Tris, pH 6.8 buffer containing 20% sodium dodecyl sulfate (SDS) along with ethylenediaminetetraacetic acid (EDTA)-free protease and phosphatase inhibitors (Thermo Fisher Scientific, USA). Samples were processed as described previously [42], and the protein concentrations were determined using bicinchoninic acid (BCA) protein assay (Thermo Fisher Scientific, USA). About 30 µg of protein from each treatment group were loaded onto a 12% or 15% SDS-polyacrylamide gel and resolved by electrophoresis. Proteins were then transferred onto polyvinylidene difluoride (PVDF) membranes (Merck GmbH, Darmstadt, Germany). The membranes were blocked with 5% nonfat milk or bovine serum albumin (BSA) (Sigma-Aldrich, Hamburg, Germany) for 1 h, and then probed with specific primary antibodies (overnight at 4 °C) for the following: Phospho-ERK1/2 (Thr202/Tyr204) (E10), ERK1/2, phospho-eIF2α (Ser51) (D9G8) XP^®^, E-cadherin (4A2), phospho-SMAD2 (Ser465/467) (138D4), SMAD2 (D43B4), SMAD4 (D3MU), phospho-4E-BP1 (Thr37/46) (236B4), and phospho-p70 S6 kinase (Thr389) (108D2) obtained from Cell Signaling Technology (Danvers, MA, USA); and anti-GRP78/BiP, anti-GADD153 (CHOP), anti-XBP-1, and anti-alpha-SMA procured from Abcam (Cambridge, UK). Subsequently, membranes were incubated with horseradish peroxidase (HRP)-conjugated goat antimouse IgG or goat antirabbit IgG secondary antibodies (Abcam) for 1 h at room temperature. The immunoreactivity was visualized with Optiblot ECL detection kit (Abcam) using FluorChem^TM^ M imaging system (Protein Simple, San Jose, CA, USA) and analyzed using AlphaView software (Protein Simple). The densitometry values were normalized to loading controls, β-actin (13E5) (Cell Signaling Technology), and Anti-Lamin B1 antibody (Abcam) and expressed as a percentage of control. 

### 2.8. Statistical Analysis

All values were calculated as a percentage of control and expressed as mean ± standard error of the mean (SEM). One-way analysis of variance (ANOVA) followed by Tukey’s post-hoc test was performed using Prism 7 (GraphPad, San Diego, CA, USA) to determine statistical differences between the various treatment groups. A *p*-value less than 0.05 was considered statistically significant. 

## 3. Results

### 3.1. High Glucose-Derived MPs Do Not Cause Cytotoxicity or Affect Cell Cycle in Naïve RPTCs

**Figure 1:**
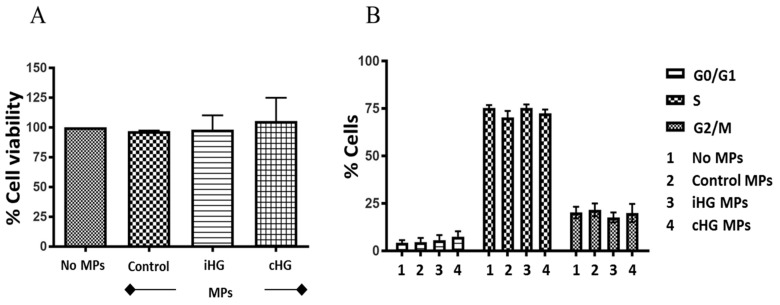
High glucose-derived microparticles (MPs; 10 µg/mL) do not affect the cell viability and cell cycle progression of naïve renal proximal tubular cells. (**A**) Cell viability determined using alamar blue assay; and (**B**) Cell cycle analysis performed using Tali^TM^ cell cycle assay kit. Values were expressed as mean ± SEM; *n* = 3–4. iHG: Intermittent high glucose; cHG: Continuous high glucose.

Intriguingly, the exposure of naïve RPTCs for 24 h to MPs generated from RPTCs stimulated with high glucose either continuously for 72 h or intermittently did not cause any changes in cell viability as measured by Alamar blue assay (Figure 1A). Our findings reveal that MPs derived from RPTCs postexposure to high glucose were not directly cytotoxic to naïve RPTCs.

Next, we investigated the effects of MPs derived from RPTCs exposed continuously and intermittently to high glucose for 72 h on the various stages of the cell cycle in naïve RPTCs. Similar to our findings from cell viability assay, no significant alterations were observed with any of the three stages of the cell cycle in naïve RPTCs incubated with MPs for 24 h (Figure 1B). These results indicate that MPs released from RPTCs in response to high glucose-induced stress do not affect the cell cycle progression in naïve RPTCs.

### 3.2. Continuous High Glucose-Derived MPs Activate mTOR Pathway in Naïve RPTCs

**Figure 2:**
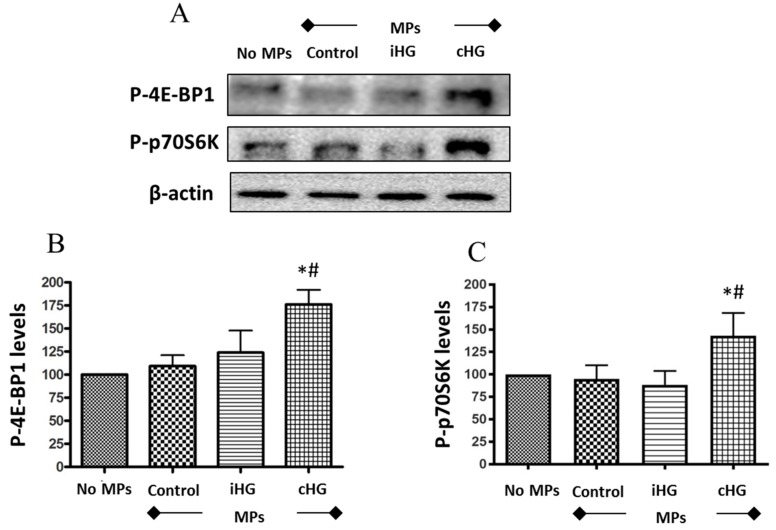
High glucose-derived microparticles (MPs; 10 µg/mL) induce phosphorylation of two downstream targets of the mammalian target of rapamycin (mTOR) pathway in naïve renal proximal tubular cells. (**A**) Representative Western blots of P-4E-BP1 and P-p70S6K; and (**B**,**C**) densitometry analyses of P-4E-BP1 and P-p70S6K levels normalized to β-actin respectively. Values were expressed as mean ± SEM; *n* = 3; * *p* < 0.05 versus no MPs (control); # *p* < 0.05 versus control MPs; P-4E-BP1: Phosphorylated-eukaryotic translation initiation factor 4E-binding protein 1; P-p70S6K: Phosphorylated-70kD ribosomal protein S6 kinase; iHG: Intermittent high glucose; cHG: Continuous high glucose.

We analyzed two downstream markers of the mTOR pathway—phosphorylated forms of eukaryotic translation initiation factor 4E-binding protein 1 (4E-BP1) and 70kD ribosomal protein S6 kinase (p70S6K). Naïve RPTCs treated with MPs isolated from the RPTCs that were continuously exposed to high glucose for 72 h showed a significant increase in the phosphorylation of both proteins-4E-BP1 (about 1.75-fold increase) (Figure 2A,B) and p70S6K (about 1.3-fold increase) (Figure 2A,C) as compared to those treated with MPs from RPTCs exposed to normal glucose (i.e., control MPs). Intriguingly, no significant increases in the phosphorylation of p70S6K and 4E-BP1 were seen in RPTCs exposed to MPs derived from RPTCs subjected to intermittent high glucose compared to the control MPs group. Together, these results suggest that the mTOR pathway was activated in naïve RPTCs by MPs shed from RPTCs exposed continuously to high glucose, which might contribute to excessive renal cell proliferation and hypertrophy. 

### 3.3. High Glucose-Derived MPs Activate ERK Pathway in Naïve RPTCs

**Figure 3:**
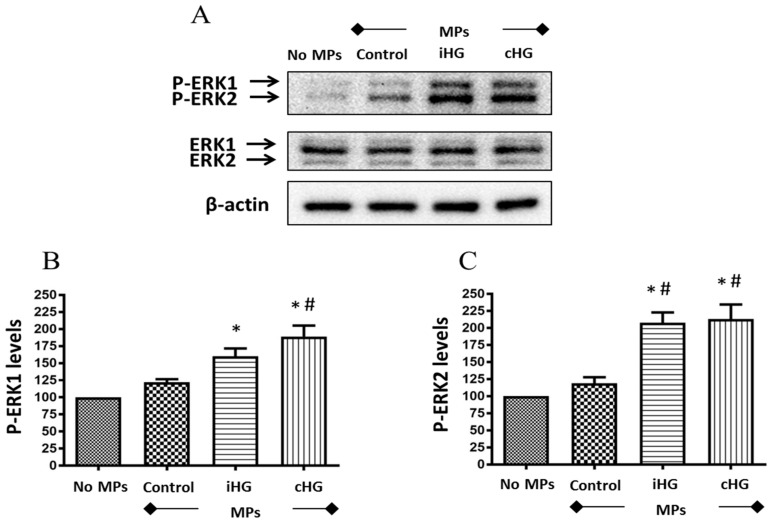
High glucose-derived microparticles (MPs; 10 µg/mL) induce phosphorylation of the downstream target of extracellular signal-regulated kinase (ERK) pathway—ERK1/2—in naïve renal proximal tubular cells. (**A**) Representative Western blot of P-ERK1/2; and (**B**,**C**) densitometry analyses of P-ERK1 and P-ERK2 levels normalized to β-actin. Values were expressed as mean ± SEM; *n* = 3; * *p* < 0.05 versus no MPs (control); # *p* < 0.05 versus control MPs; P-ERK1/2 and ERK1/2: Phosphorylated and total extracellular signal-regulated kinases 1/2; iHG: Intermittent high glucose; cHG: Continuous high glucose.

To ascertain whether MPs trigger signaling pathways involved in the control of cell proliferation, we assessed the impact of high glucose-derived MPs on the activation of ERK pathway by specifically assessing the phosphorylation of extracellular signal-regulated kinases—ERK1/2—in the naïve RPTCs exposed to MPs. Our results revealed that MPs derived from RPTCs exposed to high glucose (intermittent and continuous) increased the phosphorylation of ERK1/2—about 1.55-fold increase in intermittent high glucose treatment and about 1.8-fold increase in continuous high glucose treatment as compared to the untreated control, i.e., no MPs-treated group (Figure 3A–C). Moreover, a small increase in the phosphorylation of ERK1/2 (about 1.2-fold increase) was seen in naïve RPTCs treated with MPs from control RPTCs as compared to untreated control RPTCs, which indicate that MPs from healthy RPTCs tend to activate ERK1/2 signaling in naïve RPTCs.

### 3.4. High-Glucose Derived MPs Partially Activate ER Stress in Naïve RPTCs 

**Figure 4:**
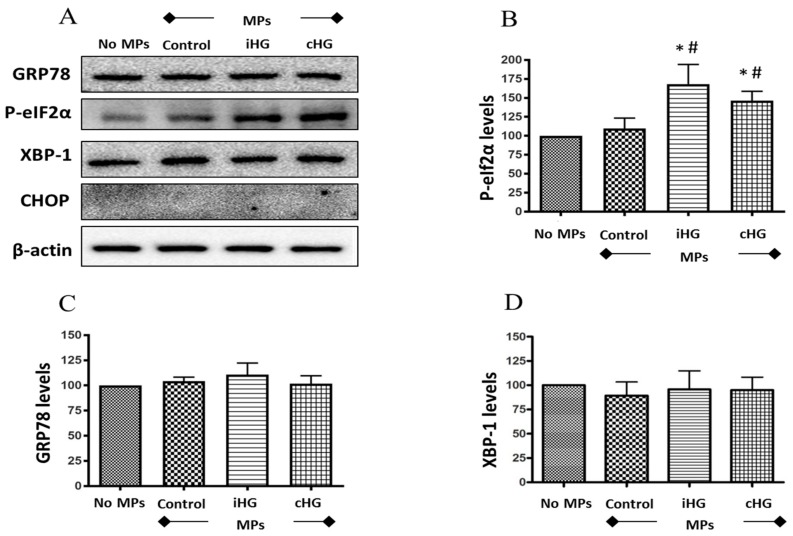
High glucose-derived microparticles (MPs; 10 µg/mL) induce phosphorylation of eIF2α in naïve renal proximal tubular cells. (**A**) Representative Western blots of endoplasmic reticulum (ER) stress markers - GRP78, P-eIF2α, XBP-1, and CHOP; and (**B**–**D**) densitometry analyses of P-eIF2α, GRP78, and XBP1 levels normalized to β-actin. Values were expressed as mean ± SEM; *n* = 3; * *p* < 0.05 versus no MPs (control); # *p* < 0.05 versus control MPs; GRP78: Glucose-regulated protein 78; P-eIF2α: Phosphorylated-eukaryotic initiation factor 2α; XBP-1: X-box binding protein 1; CHOP: CCAAT-enhancer-binding protein homologous protein; iHG: Intermittent high glucose; cHG: Continuous high glucose.

We studied the effect of MPs on the expression of ER stress markers such as phosphorylated- eukaryotic initiation factor 2α (P-eIF2α; Ser51), glucose-regulated protein 78 (GRP78), X-box binding protein 1 (XBP-1), and CCAAT-enhancer-binding protein homologous protein (CHOP). Of the four markers of ER stress we had examined, exposure of naïve RPTCs to MPs derived from RPTCs both intermittently and continuously treated with high glucose only affected the expression levels of P-eIF2α (Ser51) (about 1.7-fold increase over the control), which is an early marker of ER stress (Figure 4A,B). We observed no changes in the expression of the other ER stress markers such as GRP78 and XBP-1, and the expression of CHOP was undetectable in the untreated as well as all three MPs-treated RPTCs (Figure 4A–D). These data suggest that high glucose-derived MPs may contribute to the transient inhibition of protein translation in naïve RPTCs given the critical role of P-eIF2α in controlling this action.

### 3.5. Intermittent High Glucose-Derived MPs Activate the TGF-β Pathway in Naïve RPTCs

**Figure 5:**
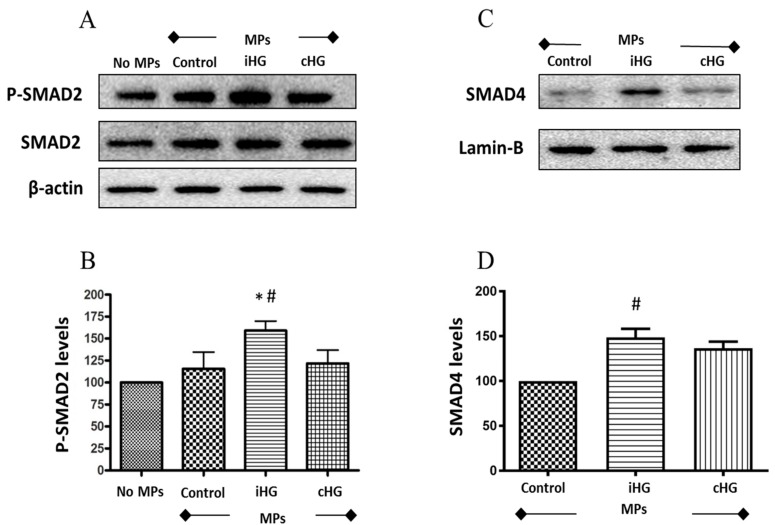
High glucose-derived microparticles (MPs; 10 µg/mL) activate transforming growth factor-beta (TGF-β) pathway via phosphorylation of SMAD2 and nuclear translocation of SMAD4 in naïve renal proximal tubular cells. (**A**) Representative Western blot of phosphorylated SMAD2; (**B**) densitometry analysis of P-SMAD2 levels normalized to β-actin; (**C**) representative Western blot of SMAD4 in the nuclear fraction; and (**D**) densitometry analysis of SMAD4 levels normalized to Lamin-B. * *p* < 0.05 versus no MPs (control); # *p* < 0.05 versus control MPs; P-SMAD2: Phosphorylated-SMAD2; iHG: Intermittent high glucose; cHG: Continuous high glucose.

To tease out the effects of MPs (derived from high glucose-treated RPTCs) on the activation of TGF-β signaling in naïve RPTCs, we studied two important markers of TGF-β pathway—SMAD2 and SMAD4 proteins. We observed a 1.6-fold increase in the phosphorylation of SMAD2 protein in naïve RPTCs treated with MPs from RPTCs exposed to intermittent high glucose compared to the other two MPs-treated groups (Figure 5A,B). This increase in SMAD2 phosphorylation was also accompanied by increases in the levels of SMAD4 (about 1.4-fold increase) in the nuclear fraction of RPTCs exposed to MPs isolated from RPTCs exposed to intermittent high glucose as compared to its concurrent control (Figure 5C,D). Collectively, these findings indicate that MPs derived from RPTCs exposed intermittently to high glucose caused activation of the TGF-β pathway in naïve RPTCs.

### 3.6. High Glucose-Derived MPs Induce EMT in Naïve RPTCs

**Figure 6:**
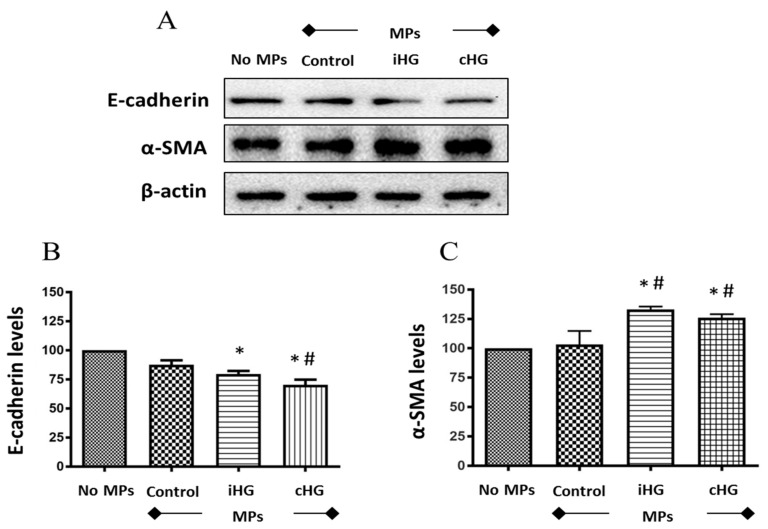
High glucose-derived microparticles (MPs; 10 µg/mL) induce epithelial-mesenchymal transition (EMT)—demonstrated by a decrease in epithelial-cadherin (E-cadherin) levels and increase in alpha smooth muscle actin (α-SMA) levels—in naïve renal proximal tubular cells. (**A**) Representative Western blots of EMT markers—E-cadherin and α-SMA; and (**B**,**C**) densitometry analyses of E-cadherin and α-SMA expression normalized to β-actin, respectively. Values were expressed as mean ± SEM; *n* = 3; * *p* < 0.05 versus no MPs (control); ^#^ *p* < 0.05 versus control MPs; E-cadherin: Epithelial-cadherin; α-SMA: Alpha smooth muscle actin; iHG: Intermittent high glucose; cHG: Continuous high glucose.

We analyzed the expression of two critical markers of the EMT pathway, i.e., E-cadherin (an epithelial marker) and alpha smooth muscle actin (α-SMA; a mesenchymal marker) in RPTCs exposed to high glucose-generated MPs. We observed significant reductions in the expression of E-cadherin (about 1.3-fold decrease) in naïve RPTCs exposed to MPs generated from RPTCs exposed continuously or intermittently to high glucose as compared to the untreated control RPTCs (Figure 6A,B). Moreover, a concomitant induction of mesenchymal marker α-SMA (about 1.3-fold increase) was noted in the naïve RPTCs exposed to MPs obtained from high glucose-treated RPTCs as compared to those treated with MPs from the control group (Figure 6A,C). 

## 4. Discussion

### 4.1. Cell Viability and Cell Cycle Progression

There are no studies, to the best of our knowledge, that have studied the cytotoxic effects of MPs shed in response to high glucose-induced stress on RPTCs. So, we compared the effects of MPs isolated from the RPTCs exposed for 72 h to normal glucose and high glucose conditions (intermittent and continuous) on the viability and the cell cycle progression of naïve RPTCs. Our findings reveal that MPs shed by RPTCs in response to high glucose stress neither affects the viability nor the cell cycle progression of naïve RPTCs. As the contents of the MPs are largely dependent on the originating cell and the nature of stimuli, our findings suggest that RPTCs exposed to high glucose conditions do not shed MPs containing cytotoxic or antiproliferative signals.

### 4.2. mTOR Pathway

The mTOR signaling pathway stimulates cell growth, survival, and proliferation. We observed a significant increase in the phosphorylation of two crucial downstream targets of the mTOR pathway—4E-BP1 and p70S6K. The function of unphosphorylated 4E-BP1 is to bind to the eukaryotic translation initiation factor 4E (eIF4E) and inhibit mRNA processing. However, when it is phosphorylated, 4E-BP1 cannot bind to eIF4E, and thus, the mRNA translation is uninhibited. Similarly, the function of phosphorylated p70S6K is to enhance the synthesis of proteins that are essential for cell growth and proliferation. Our study revealed that both downstream markers of mTOR—4E-BP1 and p70S6K—were phosphorylated only in the presence of MPs obtained from RPTCs that were continuously treated with high glucose for 72 h. These findings indicate that mTOR signaling was activated by MPs shed by RPTCs under sustained hyperglycemic conditions, which may contribute to the hypertrophy of renal tubular cells and buildup of fibrotic tissue, which represents a hallmark of DN [43,44]. Since decreased protein degradation in renal tubules was also shown to contribute to diabetic renal hypertrophy [45], future studies that examine the effect of high glucose-derived MPs on proteolytic pathways including autophagic-lysosomal protein degradation in RPTCs are warranted.

It is also important to note that the activation of mTOR is in divergence to our findings with TGF-β, where the activation was seen only in naïve RPTCs treated with MPs generated from cells treated with intermittent high glucose. These data highlight the importance of fluctuations in glucose levels, a situation commonly observed during diabetes, in the differential control of the expression of growth factors and other signaling molecules secreted into MPs under high glucose stress. This further underscores the importance of the stimulus at the origin of MP shedding in conditioning the cargo content of vesicles as well as the biological messages carried out by MPs [15]. 

### 4.3. ERK Pathway

In further support to our findings from the markers of mTOR pathway, our results show a profound increase in the phosphorylation of ERK1/2 following the treatment of naïve RPTCs with MPs obtained from RPTCs exposed to high glucose for 72 h—whether it was intermittent or continuous. In corroboration to our findings, transient activation of p38 mitogen-activated protein kinase (MAPK), a signaling pathway closely related to ERK1/2, was demonstrated in cultured human proximal tubule cells following exposure to MPs derived from podocytes [21]. Since the ERK pathway is activated in the presence of growth factors and cytokines, the activation of ERK1/2 following treatment with MPs provides indirect evidence that MPs shed from RPTCs post-high glucose stress carry a cargo of growth factors, which can induce cell proliferation. 

### 4.4. ER Stress Response

Several studies indicate that ER stress plays a major role in the pathogenesis of DN [46,47,48,49,50]. We observed a steady increase in the phosphorylation of eIF2α in naïve RPTCs in the presence of MPs isolated from RPTCs exposed intermittently or continuously to high glucose. In the PERK pathway of ER stress, phosphorylation of eIF2α was the first event to occur, and this often follows ER stress. However, the initiation of this event cannot be misconstrued as ER stress. Intriguingly, there were no significant changes in the expression of other proteins involved in ER stress pathway, namely GRP78, XBP-1, and CHOP. Previously, Safiedeen et al. [51] showed that MPs from apoptotic T-cells and metabolic syndrome patients increased the levels of ER stress markers—P-eIF2α, XBP-1, and CHOP—in human aortic endothelial cells [51]. Our study is the first of its kind to have examined the paracrine effects of MPs derived from RPTCs in triggering ER stress in naïve RPTCs. Our findings suggest that MPs from RPTCs exposed to high glucose do not evoke an ER stress response in naïve RPTCs, although they can trigger the phosphorylation of eIF2α and may, therefore, cause a transient reduction in the translation of proteins. 

### 4.5. TGF-β Pathway

In this study, we examined two critical downstream markers of TGF-β pathway—SMAD2 and SMAD4—involved in the progression of renal fibrosis in DN [52,53]. Following the activation of the TGF-β pathway, SMAD proteins—SMAD2 and SMAD3—undergo phosphorylation and form a complex with SMAD4. The resulting trimeric complex translocates to the nucleus and modulates the transcription of genes involved in fibrotic signaling response [53]. To investigate the cascade of the events downstream of SMAD2 phosphorylation, we divided the proteins into cytosolic and nuclear fractions. p-SMAD2 expression was analyzed in the cytosolic fraction, and SMAD4 expression was analyzed in the nuclear fraction as SMAD4 expression was undetectable in the cytosolic fraction. 

Consistent with our findings from the EMT pathway, we noted an increase in the expression of both markers of TGF-β. Specifically, we observed a profound increase in the phosphorylation of SMAD2 and the levels of SMAD4 (in the nuclear fraction) in naïve RPTCs treated with MPs generated from cells exposed to intermittent high glucose as compared to that of those from continuous high glucose treatment. It is intriguing that no changes in the phosphorylation of SMAD3 protein were observed in any of the groups (data not shown). Our findings are consistent with observations that have been previously documented in studies using exosomes derived from RPTCs in response to hypoxic stimuli [54] and from glomerular cells subjected to high glucose stress [55,56]. For example, Borgers et al. [54] showed that exosomes derived from RPTCs subjected to hypoxic stress promote renal fibrosis via induction of TGF-β1 and α-SMA expression in renal fibroblasts [54]. Other studies also demonstrated that exosomes derived from high glucose-treated glomerular endothelial cells [55] and glomerular mesangial cells [56] activate TGF-β1 signaling in podocytes. Together, our findings suggest a strong profibrotic role of MPs derived from RPTCs under high glucose conditions. 

### 4.6. EMT Pathway

Numerous studies implicate the role of EMT in the development of renal fibrosis [33,34,57,58,59,60]. During the process of EMT, cells lose cell adhesion proteins specific to epithelial phenotype, such as E-cadherin, and start to express proteins that are specific to that of fibroblast phenotype, such as α-SMA. Hence, we studied the expression of two critical markers of EMT: E-cadherin and α-SMA, in the naïve RPTCs postexposure to MPs. Our findings revealed that the expression of E-cadherin was decreased in naïve RPTCs in the presence of MPs obtained from RPTCs exposed to high glucose. Simultaneously, we observed an increase in the mesenchymal marker α-SMA in the presence of MPs derived from RPTCs exposed to high glucose conditions. Studies have shown that MPs derived from podocytes, endothelial cells, and RPTCs are biomarkers of DN and also mediate renal fibrosis [21,22]. Wu et al. showed that exosomes derived from high glucose-treated glomerular endothelial cells induce α-SMA expression in podocytes and glomerular mesangial cells [55,61]. Together, these studies suggest that MPs and exosomes mediate cell-to-cell communication in the progression of renal fibrosis in DN. In corroboration with the findings on exosomes [55,61], our study indicates that MPs shed by RPTCs subjected to sustained high glucose stress trigger naïve renal cells to undergo the transition from epithelial to fibroblast phenotype. In addition, our findings also suggest that these MPs might contain growth factors and cytokines, which could signal distant healthy RPTCs to undergo EMT and potentially, fibrosis of the kidney. Additional studies are warranted to understand the specific role of MPs as mediators of EMT in DN.

## 5. Conclusions

**Figure 7:**
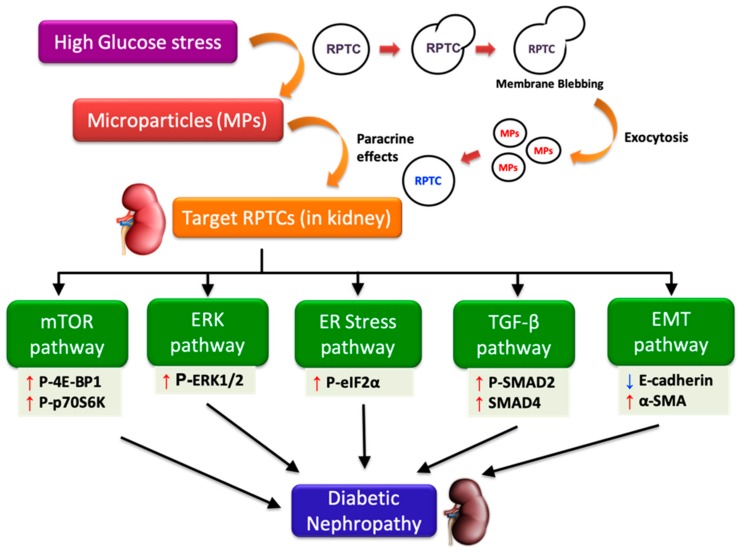
High glucose-derived microparticles (MPs; 10 µg/mL) induce multiple signaling pathways such as the mammalian target of rapamycin (mTOR), extracellular-signal regulated kinase (ERK), endoplasmic reticulum (ER) stress, transforming growth factor-beta (TGF-β), and epithelial-mesenchymal transition (EMT) in renal proximal tubular cells (RPTCs), and might potentially contribute to the development of diabetic nephropathy. Upward and downward arrows, respectively, indicate increase and decrease in protein levels. P-4E-BP1: Phosphorylated-eukaryotic translation initiation factor 4E-binding protein 1; P-p70S6K: Phosphorylated-70kD ribosomal protein S6 kinase; ERK1/2: Extracellular-signal regulated kinase 1/2; P-eIF2α: Phosphorylated-eukaryotic initiation factor 2α; P-SMAD2: Phosphorylated-SMAD2; E-cadherin: Epithelial-cadherin; α-SMA: Alpha smooth muscle actin.

Although previous studies have reported the role of MPs from cell types such as endothelial cells [18,51], platelets [62], and podocytes [20,21] in DN and chronic kidney disease [51,63,64], the effects of MPs shed by RPTCs under high glucose conditions on neighboring RPTCs is largely unknown. The present study indicates that under hyperglycemic conditions, MPs released from RPTCs activate several pathways such as mTOR, ERK, TGF-β, and EMT, in neighboring RPTCs and contribute to the progression of DN (as depicted in Figure 7). Our studies also suggest that intermittent and continuous high glucose in RPTCs lead to the production of MPs of more or less a similar composition, reflected by the comparable activation of several signaling pathways in naïve RPTCs. However, we did observe that only the MPs derived from continuous high glucose activated mTOR pathway, and only those derived from intermittent high glucose activated TGF-β pathway. The precise mechanisms that attribute to these deviations need further investigation; however, these discrepancies underscore the complexity of MP signaling and the importance of the stimulus causing the release of MPs in conditioning the cargo content of the shed MPs in addition to the biological messages that they vehicle. Further studies to investigate the involvement of other mechanisms implicated in the progression of DN, such as oxidative stress [10,18] and fibrosis [21], would shed more light on the mechanisms that underlie MP-induced renal cell injury.

Our study extends the knowledge on the potential mechanisms involved in the progression of renal fibrosis in DN by elucidating that MPs released by RPTCs under high glucose conditions play a critical role in communicating the signals from stressed RPTCs to naïve RPTCs through paracrine signaling. It is noteworthy to mention that elevated levels of circulating MPs have been shown to serve as an independent marker of the cardiovascular disease and high mortality observed in diabetes patients with ESRD undergoing hemodialysis [15,65]. Taken together with our study findings, MPs derived from renal tubular cells might also contribute to the increased mortality observed in diabetes patients receiving hemodialysis. Thus, investigating the role of MPs-mediated cell-to-cell communication in future studies would be helpful to design new, effective therapeutic strategies to attenuate the progression of DN in patients and to decrease mortality in diabetes patients with ESRD.
